# Developing evaluation capacities in integrated care projects: Lessons from a scientific support mission implemented in Belgium

**DOI:** 10.3389/fpubh.2022.958168

**Published:** 2022-11-15

**Authors:** Nathan Charlier, Elien Colman, Lucia Alvarez Irusta, Sibyl Anthierens, Thérèse Van Durme, Jean Macq, Benoit Pétré

**Affiliations:** ^1^Department of Public Health, University of Liège, Liège, Belgium; ^2^Department of Family Medicine and Population Health, University of Antwerp, Antwerpen, Belgium; ^3^Institute of Health and Society, Université Catholique de Louvain, Brussels, Belgium

**Keywords:** evaluation capacity, evaluation capacity building (ECB), integrated care programs, information-based approach, scientific support, decision making, applied research, learning community

## Abstract

The capacity of self-assessment, to learn from experience, to make information-based decisions, and to adapt over time are essential drivers of success for any project aiming at healthcare system change. Yet, many of those projects are managed by healthcare providers' teams with little evaluation capacity. In this article, we describe the support mission delivered by an interdisciplinary scientific team to 12 integrated care pilot projects in Belgium, mobilizing a set of tools and methods: a dashboard gathering population health indicators, a significant event reporting method, an annual report, and the development of a sustainable “learning community.” The article provides a reflexive return on the design and implementation of such interventions aimed at building organizational evaluation capacity. Some lessons were drawn from our experience, in comparison with the broader evaluation literature: The provided support should be adapted to the various needs and contexts of the beneficiary organizations, and it has to foster experience-based learning and requires all stakeholders to adopt a learning posture. A long-time, secure perspective should be provided for organizations, and the availability of data and other resources is an essential precondition for successful work.

## Introduction

In Europe, many policy programs aim at reforming health system as a mean to respond to interconnected challenges, such as rising costs, an aging population, the growing burden of chronic disease, or patient centeredness, among other (1). Many of these programs explicitly or implicitly pursue the triple (2) or quadruple aim (3) as an overarching goal, that is, improving population health, improving patient experience, reducing costs, and improving healthcare providers' work experience.

Integrated care (IC) is often presented as a solution to achieve the quadruple aim. It can be defined as “an approach to strengthen people-centered health systems through the promotion of the comprehensive delivery of quality services across the life-course, designed according to the multidimensional needs of the population and the individual, and delivered by a coordinated multidisciplinary team of providers working across settings and levels of care. It should be effectively managed to ensure optimal outcomes and the appropriate use of resources based on the best available evidence, with feedback loops to continuously improve performance and to tackle upstream causes of ill health and to promote wellbeing through intersectoral and multisectoral actions” (4).

Many IC initiatives have been implemented and extensively studied in Europe over the past two decades: The academic literature is abundant regarding key factors and conditions leading to successful implementation of IC initiatives (5–7). Many of these factors are related to organizational capacities (8–10), as IC initiatives usually entail ambitious project implementation, networking, and development of new governance structures. In this regard, the capacity of self-assessment, to make decisions based on sound information, to learn from experience, and to adapt over time are essential factors of success (11, 12) for IC projects and for any project aiming at health system change. IC project implementation and governance usually involve several healthcare providers and services; however, these actors are not necessarily trained to monitor and evaluate their project's results over time. Therefore, such initiatives are often accompanied by academics to support project management and evaluation (13): Scientific accompaniment of involved actors is a way to develop organizational capacity to implement, govern, and evaluate ambitious action plans. This accompaniment can be labeled as a “capacity building intervention” (14, 15).

We, the authors, have designed and implemented such a capacity building intervention to support and train 12 integrated care pilot projects (ICPs) in Belgium between January 2018 and June 2020. This article, a practice review, provides a reflexive return on our experience with this mission. Reporting our work, detailing the lessons we learned and elaborating specific recommendations in relation with the broader evaluation literature can feed future researchers, policymakers, and practitioners to engage in capacity building activities dedicated to interdisciplinary care providers' teams. It also contributes to the growing literature about capacity building intervention and evaluation capacity building in the healthcare domain.

The information we present in the article comes from multiple data sources. Primarily, our support work was developed alongside an evaluation mission we were in charge of: We evaluated the implementation of the 12 ICPs and their achievements over time. The protocol of this mixed-method, realist evaluation is detailed in another article (16). The evaluation of ICPs' implementation focused, among other things, on governance mechanisms, and on quality culture and self-evaluation practices developed within the ICPs. Therefore, we collected diverse qualitative data to feed our evaluation: Three focus groups were organized with key stakeholders from three different ICPs (project managers and partners involved in project governance), an online questionnaire with open-ended and closed questions regarding self-evaluation was submitted to members of the 12 ICPs consortia, and we performed a document analysis of each projects' action plan and annual reports. Furthermore, the account of our work presented in the current article is also fed by several activity traces and documents we produced: the general, peer reviewed protocol, meeting minutes, internal operational documents to guide our consortium members, and documents about our mission that were communicated to the ICPs and to policymakers. Even though our capacity building intervention was not initially designed as a research project with a dedicated methodology, the evaluation we performed allowed to assess some of the effects of our support work at ICPs' level. Based on our experience and numerous interactions with the ICPs and with policymakers, we were able to understand whether the tools and methods we had developed were adopted and how the messages we had delivered were received.

The article is structured as follow: First, the policy program funding the 12 pilot projects and our support and evaluation mission will be presented, as well as the Belgian contextual specificities leading to this care integration reform. Then, the set of methods and tools that were designed and implemented to support the pilot projects and train their teams to self-evaluation will be presented, as well as some achievements and practices we could identify at ICPs' level. Each subsection will detail the design and implementation of one capacity building tool or method. Finally, the discussion will provide further reflections, lessons learned, and recommendations on how to develop and implement evaluation capacity building interventions applied to healthcare, in relation with the broader evaluation literature. Indeed, effectively training and supporting healthcare providers to develop information-based decision-making and to juggle with diverse evaluation methods is key, as these represent essential capacities in health system governance and transformation.

## “Integrated care for better health”: A policy program in Belgium

The number of patients with multiple chronic conditions is increasing in Belgium, in direct relation with an aging population (1, 17). Multimorbid patient care requires inputs from different providers. However, the Belgian healthcare system is mainly focused on acute diseases, with a high degree of specialization resulting in organizational fragmentation of services and a lack of coordination between care providers (18, 19): Interactions are limited between hospital specialists, general practitioners, and other primary care providers, such as pharmacists, physiotherapists, and home care nurses. This lack of interprofessional collaboration has detrimental consequences for multimorbid patients, whose care pathway and experience suffer disruptions. Consequently, their care needs remain poorly addressed. To tackle this complex challenge, the Belgian Government launched a large policy program called “Integrated Care for Better Health” in 2015 (20). The program has the ambition to achieve the “quadruple aim” (3). Integrated care is the mean to achieve this overarching goal. Yet, how to concretely develop integrated care “in the Belgian-specific context was more uncertain. This explains why the Belgian authorities decided to use pilot projects as implementation instruments” (21). Using pilot projects is one way to involve diverse care providers and services in the development of integrated care reforms, without imposing a “one size fits all” solution. This flexibility also allows to adapt the reform to diverse loco-regional contexts: Sub-regions of Belgium differ regarding the degree of urbanization, demography, socio-economic conditions, the availability of care services, and regional legislations as Belgium is a federal state. To guide the pilot projects, the policy program defined a series of 14 “components” (see Table 1) that should be pursued by the pilot projects. The policy's underlying hypothesis is that implementing actions contributing to these components would lead to integrated care.

**Table 1:** Fourteen components of integrated care framing the 12 ICPs work.

**Around the patient**
1) Empowerment of the patient
2) Support for informal caregivers
3) Case management
4) Work conservation, socio-professional and socio-educational reintegration
**Around professionals**
5) Prevention: Involving several areas
6) Consultation and coordination
7) Intra- and transmural care continuity
8) Valorisation of the experiences of patient organizations
9) Integrated patient files
10) Multidisciplinary guidelines
**At loco-regional level**
11) Development of a quality culture
12) Adaptation of the financial system
13) Stratification of the risks within the population and mapping of the area
14) Change management

After a selection process, the government decided to fund 12 integrated care pilot projects to test and implement different modalities of care integration at loco-regional level: “ICPs are both large-scale implementation projects and test cases for future scaling-up to the entire country” (16). The 12 ICPs first went through a conceptualization phase (2016–2017), during which each project had to build a network of involved health and social care organizations and to draft a broad action plan to integrate care and to achieve results regarding the program's 14 components, with the quadruple aim as an end. The implementation phase runs from 2018 until the end of 2022. Each ICP covers a geographical region between 75,000 and 360,000 inhabitants, and their network assembles a diverse set of actors, including general practitioners, pharmacists, hospitals, home care, and social workers. ICPs generally focus on a subset of their territory's population, mostly targeting people living with chronic conditions. They implement a large set of actions at different levels to re-organize healthcare provision in accordance with their target population's needs and the local context and challenges. These numerous actions are very diverse, ranging from interprofessional medication review to tackle polypharmacy, to the implementation of a “neighborhood” professional case manager to help patients to navigate in the complex care system. ICPs receive a fixed funding of 150.000 euros/year, and an extra fluctuating sum defined according to their achievements, allowing them to fund innovative interventions. With these characteristics, ICPs' consortia have developed a local health system governance. ICPs are managed by one or two coordinators, accompanied in their mission by an “executive board,” generally composed from five to 10 members that represents diverse healthcare professions or institutions. Most of the coordinators and executive board members are not trained or do not have former experience in project management and program evaluation.

The authorities made it a requirement for the ICPs to develop their self-evaluation abilities. Three components of the policy program directly refer to this issue: “Development of a quality culture,” “change management,” and “Stratification of the risks within the population and mapping of the area” to plan activities based on population needs. Therefore, the projects had to plan data collection to assess whether their objectives are met or not, following PDSA cycles (Plan-Do-Study-Act) (22). Their action plan had to include SMART (specific, measurable, achievable, realistic, time-bound) objectives and related indicators (23). If these concepts are widely accepted in quality management and program evaluation, their implementation by healthcare professionals in specific projects remains a challenge (24–26).

Therefore, the policy program planned that these ICPs should be evaluated and supported by a scientific team. A scientific interdisciplinary team called FAITH.be (Federated consortium for the Appraisal of Integrated care Teams in Health in Belgium), of which the authors are part, was selected to develop this double mission. FAITH.be protocol for a mixed-methods realist evaluation has been thoroughly described in a dedicated article (16); the present article deals with the support mission delivered by the scientific team to the ICPs. The support activities were designed to feed FAITH.be evaluation mission, since they allowed to gather specific data about each of the 12 running ICPs. In exchange, our evaluation results were fed back to the ICPs, at various points in time. The intent was to develop opportunities for the stakeholders to adapt the way they work toward care integration and for FAITH.be to further refine its analysis by receiving feedback and specific demands. Thus, the two missions, evaluation of, and support to the ICPs, should not be understood as isolated blocks, but rather as interconnected elements of a virtuous circle.

## Design and implementation of FAITH.be support mission

### Tenets and key concepts guiding the design of the support mission

The support delivered to the ICPs by FAITH.be has essentially been methodological, so that pilot projects could develop their self-evaluation capacities and become autonomous once FAITH.be mandate ended. When we designed the protocol regarding the support, we did not mobilize the capacity building intervention literature as we did not know about this specific concept. However, some key concepts derived from quality management literature and from evaluation literature guided our work, as well as similar projects developed elsewhere (27–29): First, we trained the ICPs to implement PDSA cycles, a central notion for quality culture (22) that consists in learning “whether an intervention works in a particular setting and to making adjustments accordingly to increase the chances of delivering and sustaining the desired improvement” (26). This meant that the projects should learn from their experience and should be able to find and interpret relevant data about their population's characteristics and needs to adapt the intervention plan and to foster information-based decision-making (30). Second, we promoted a pragmatic approach, according to which ICPs' teams should use as much as possible existing data, like routinely collected health insurance or hospitals data: Rather than investing in time-consuming data collection procedures, ICPs' decision makers and coordinators should first learn to analyze and interpret those available data. This leads us to the third key guiding concept: To choose relevant data and to engage with their interpretation, the pilot project should be able to elicit the “theory of change” underlying their action plan. Indeed, ICPs, with their vast and ambitious action plans, are aiming to implement complex whole system changes that cannot be reduced to simple interventions. In such cases, theory-driven evaluation can help to understand how and why a program work (31). The theory of change “describes how a program brings about specific long-term outcomes through a logical sequence of intermediate outcomes (and actions)” (32), and it is embedded in the realist evaluation paradigm (33) adopted by FAITH.be.

### Implementation of support tools and methods

In the initial protocol, FAITH.be had planned to elaborate four complementary tools to foster ICPs' information-based decision-making in a PDSA logic:

1. A dashboard gathering key indicators for population management and project monitoring.
2. A significant event reporting method to learn from experience.
3. An annual report focusing on specific questions in a series of key domains.
4. The development of a sustainable “learning community” (34) gathering the 12 ICPs' coordinators to exchange and learn from their respective experiences.

These tools pertain to different types of data, both qualitative and quantitative. For the ICPs to appropriate these tools and to help them implement self-evaluation practices leading to quality culture and PDSA cycles, the scientific team met several times with the 12 ICPs teams, either project-per-project or in a collective manner. Collective sessions were the occasion to deliver presentations and/or to make exercises about specific subjects, to present and discuss the four aforementioned tools. Project-per-project support was adapted to the ICPs' specificities: Each ICP was met at least four times between 2018 and 2020, to work on the theory of change, to carry out an exercise about population management, or to help with the completion of the annual report. Furthermore, the FAITH.be team was also available as an “on-demand helpdesk” for any question related to self-evaluation and quality culture *via* emails, phone, and face-to-face meeting. To delve into the support mission, we can briefly describe the main characteristic of the four tools and their implementation.

#### Dashboard and indicator selection

The dashboard was supposed to gather and aggregate several data sources into relevant indicators that could be followed over time, per ICP area. This would have helped ICPs' teams to summarize information and analyze data, interpret results, identify trends, and draw conclusions by monitoring the progress of a series of key indicators related to the characteristics of the population changes in healthcare provision, costs, and other measurable outcomes (35). However, such a dashboard has not been delivered in a workable format, partially due to administrative fragmentation. Indeed, the definition, collection, and availability of data involved the collaboration of numerous stakeholders (FAITH.be scientific consortium, sickness funds, public authorities, IT collaborators, etc.). The construction of the “data warehouse” assembling the multiple health databases has been very complex for technical reasons, as these pre-existing databases were designed by different parties, with incompatible structural features. Furthermore, the agency in charge of developing the dashboard did not have access to the SAS visual analytics software and experienced a high turnover in its staff. More delay was added because compliance to the newly adopted EU GDPR had to be verified. Due to these restrictions, only a proof of concept was eventually made available within the project's time frame. To overcome this absence, FAITH.be met with each of the 12 ICPs to realize exercises on population management in the form of a serious game (36), using the limited set of available data about reimbursed care provision and sociodemographic characteristics of the ICPs' populations. It is described in the Box 1.

Box 1 **Assembling existing health data to assess the results of ICPs:**

With each ICP, we identified a series of expected benefits for specific subpopulations targeted by their action plan. Based on this information, a working hypothesis was built to explain how the project expects the intervention to act and at which level (which action(s), for which population(s), generating with results?). Then, using a set of cards describing different routinely available data, the participants could assemble the cards reflecting their reasoning to develop relevant indicators which could help evaluating the effectiveness of the intervention. For example, for ICPs developing case management (37) for people with complex care needs, we were able to look at re-hospitalization patterns for specific categories of patients based on age, socio-economic status, and so on. For ICPs implementing medication reviews for poly-medicated patients, we were able to analyze the evolution of different drugs' consumption over time in specific subsets of population. The indicator construction exercise was repeated with each of the 12 ICPs, leading to a large variety of proposals.

ICPs' teams were warned of the inherent limitations and possible biases of these indicators due to the lag and nature of the data. The goal of such exercise was for ICPs' teams to learn to reflect on how to evaluate their achievements based on existing data, so that they could use a dashboard in a relevant way, following a population management approach. ICPs' partners declared they regretted the absence of the dashboard, as they could foresee the potential benefits of this tool, especially to assess trends and changes over time for a series of indicators. However, while using the indicators, some ICPs' partners highlighted that it was a complex task to choose and define the right indicators to evaluate an action. Unsurprisingly, coordinators and ICP's partners without quality management training or without epidemiology and statistical background struggled to identify and use available data as relevant indicators.

#### Significant event reporting

Significant event reporting was developed by FAITH.be as a narrative method to collect information and learn from experience, based on existing practices in healthcare institutions: Incident reporting systems initially emanated from safety management (aerospace, hospitals, high-risk facilities…), to learn from unexpected events that caused, or might have caused harm, and to avoid the reoccurrence of such problems (38). The notion of “significant event” was defined as any event, milestone, or circumstance that causes, has caused or could have caused great difficulty or, to the contrary, a leap forward in the implementation of the ICP planned activities or in the achievement of expected results/goals, and whose analysis can generate learning for the ICPs. The reporting and analysis should be done following a template of open-ended questions (see Box 2) which was adapted from various existing methodologies, to fit the ICP's context and needs. Particularly, the tool combines useful elements from “critical incident” analysis (39), “root cause analysis” (40), and the “most significant change technique” (41). One collective session was organized to sensitize ICPs' coordinators to the method: The template was exemplified *via* a concrete case about governance turnover in one ICP.

Box 2 **Template for incident reporting:**

- Date of the reporting
- Short description
- Who has been involved in the reporting
- Narrative of the event including:
  ° Actors at stake
  ° Why is this event significant?
  ° Identification of contributing factors
  ° Positive and negative consequences
  ° Timeline
- Lessons learned, recommendations would you like to share (part of) this report externally? With whom?

Even though reporting methods are now common in hospitals, to our knowledge, the significant event reporting method elaborated by FAITH.be was not used by ICPs. It has been qualified as too vague by some coordinators; it seems complex for ICP members to identify what can qualify as “significant events,” and even more to foresee the added value of the reporting and analysis process. Furthermore, some events might be related to internal tensions or conflicts between ICPs' members: This, and the fact that the reporting method was not developed with the ICPs themselves, can explain the low success of the method.

#### Annual report

The annual report follows a template focusing on a series of predefined themes (e.g., governance, inclusion, follow up of the action plan, communication), each time with open-ended questions regarding what has been achieved in the past year, what is planned for the future, and what were the possible facilitators and barriers, as perceived by the ICPs' executive boards. This template pursues a triple goal: Reporting to the authorities in a logic of accountability, providing information to the scientific team for the evaluation, and pushing the projects to reflect and assess the achievements of the previous year, to draw lessons and to decide possible adaptations. ICPs' executive boards were supposed to collectively draft answers to the questions of the template, to take time at least once a year to discuss their accomplishments and compare them to the planned operational objectives pursued by the project. The annual report completion was made mandatory by the authorities, so each ICP did provide answers about their yearly achievements. However, the accountability logic demanded by the authorities might clash with the goal of transparent self-evaluation. More specifically, at some point during the ICPs' implementation, members of the public authorities had the intention to distribute funding according to each pilot project “performance,” the latter being partially measured based on some annual report items. Furthermore, different logics and expectations were at play: Authorities expected the projects to reach many patients early on, while ICPs' teams first wanted to focus on integrating partners in the governance and developing effective communication and decision mechanisms. This generated a risk of drafting biased answers: ICPs could have chosen to present a bright face to the authorities and hide some internal difficulties and delays in the action plan implementation. We did not collect evidence demonstrating such a situation. On the contrary, many ICPs' teams did use the elaboration of the annual report as an occasion to gather their members to reflect collectively on their work, the difficulties they faced or the success they were able to achieve. In that sense, the tool was used successfully, as a mean to develop organization evaluation capacity.

#### Learning community

Finally, FAITH.be developed a “learning community” for the ICP coordinators and the main stakeholders involved in the policy program implementation. The idea was to gather stakeholders in order for them to exchange their experiences and co-construct situated knowledge about the ongoing processes (42–44). Three meetings were organized and facilitated by FAITH.be members to generate exchanges between ICPs' coordinators based on their experience and to identify and discuss good practices and lessons. Many ICPs go through similar difficulties, but develop different approaches to deal with these: Exchange can always be fruitful for pilot projects whose goal is to produce experiential knowledge about integrated care implementation. The learning community was designed to be perpetuated after the end of FAITH.be mission, *via* regular meetings and written exchanges. The first meetings were challenging, because 2018 was very early in the implementation process for ICPs. As their members did not have much concrete experience to exchange, these first meetings were the occasion to get to know one another, to answer ICPs' questions, and to discuss the first evaluation results. In 2019, with almost 2 years of actual implementation of the action plans, the coordinators now had a lot more to exchange about how things went in their ICP, what challenges they faced, what were their successes, and so on, leading to a more fruitful and bottom-up learning community. More specifically, this was the occasion to present and discuss a series of “promising practices” that FAITH.be had identified with each ICPs, and which could become drivers for care integration at loco-regional level: These promising practices were concrete instances of potentially successful (set of) actions implemented by ICP teams that could inspire other pilot projects. For example, an action developed in the rural province of Luxembourg, where there is a shortage of primary care providers, relied on the pharmacists to develop new prevention and screening services (e.g., diabetes and prediabetes screening for at risk clients), which allowed to quickly involve new patients in other care integration actions implemented by the ICP. Another example was the creation of a new “proximity referent” function. This was implemented by the ICP active in Brussels, where there is a high rate of precarious population who do not have a referent general practitioner. This population is subject to interconnected social and medical frailties: The proximity referent's role is to accompany and orient the patient toward the adequate services. The presentation of such initiative led to very fruitful collective exchanges between the participants. Beyond the formal meetings organized by FAITH.be, the learning community has been thriving, as there has been several, regular interactions between the 12 ICPs' coordinators. They have recurring discussions and exchanges, they support each other, and this has allowed them to learn from each other's experiences. One ICP also developed its own learning community to support the adaptive implementation of a specific action, involving the different stakeholders in order for them to exchange about their experiences.

#### Evaluation practices developed by ICPs

In the final evaluation of the ICPs' achievements, among other things, we analyzed whether the projects had developed information-based governance mechanisms. Eventually, we could identify that ICPs had implemented many practices pertaining to quality culture and evaluation, besides FAITH.be support tools. First, several data collection initiatives have been developed, including article or online surveys (qualitative and quantitative), focus groups, and access to local partners' quantitative data. Some of these are well established in ICP practices and the data collection is renewed repeatedly, as a mean to evaluate progress over time. Another highlighted practice is the implementation of their actions as small “pilot interventions”: Many ICPs first involve a limited number of partners to test the action and adapt it, as they expect convincing results regarding the effectiveness and appropriateness of the action before upscaling it. Finally, we could identify a facilitator for some ICPs, as they relied on the expertise of actors with specific skills and knowledge regarding quality culture and data analysis. For example, the partnering hospitals could provide ICPs with assistance regarding quantitative methods. This resulted in disparities from one ICP to another, as some teams had more expertise regarding evaluation methods than others. Overall, these practices witnessed at ICPs' level show that the PDSA logic has been well understood and adopted. ICPs' coordinators understand the importance of adapting actions according to their experiences and to the quickly evolving context, and they develop specific practices to be able to evaluate their achievements based on empirical data.

## Discussion: ICPs' quality culture and self-evaluation practices after 2.5 years

Because the timing of our mandate did not allow for it, we did not perform a comprehensive evaluation of the support mission and its outcomes at ICP level. However, we can draw some lessons based on our experience with the ICPs. Overall, the implementation of the support mission by the scientific team has been partly successful, with mixed results, as only some of the methods and tools were used by some of the pilot projects. Further research should be conducted to analyze what was retained by ICPs after a year or more without any support from the scientific team.

### Tinkering a coherent set of tools and methods

To develop the scientific support regarding self-evaluation capacities within ICPs, we had to find inspiration from a variety of sources and experiences. Quality culture and evaluation practices' implementation in specific programs and organizations are well documented in the literature (45–47). Scientific accompaniment for interdisciplinary teams in charge of managing and implementing large-scale project, such as ICPs, is quite common. However, supporting and training techniques to develop evaluation capacity within such projects are not thoroughly described and discussed in the IC literature. Therefore, we developed the initial protocol of our support mission using various sources of inspiration (28, 29, 48) and adapting it to the specific context and needs of ICPs. This resulted in a tailor-made set of practices, tools, and methods. Yet, this experience can inform scientific support and evaluation capacity building interventions elsewhere, as the evaluation capacity building research is an emerging field, with very few empirical accounts in the healthcare and care integration domains (15, 49). Some key elements characterize our support work. First, we pursued the aim of making ICPs self-sufficient in terms of evaluation and information-based decision-making. This meant there was a need for tools and methods that can be used by diverse actors without any help in the long run, once the support ended. We aimed at developing organizational evaluation capacity, rather than individual one, as there is a turnover within teams. Furthermore, the dashboard of indicators, the significant events reporting system, the annual report, and the learning community are anchored in different evaluation approaches and practices, but they are complementary: They allow to collect and analyze diversified sources of quantitative and qualitative information. They are also coherent with a certain vision of (self-)evaluation. Having an overarching evaluation framework for the support has been highlighted as a strength (49). In this case, realist theory-driven evaluation is better adapted to complex and ambitious pilot projects than quasi-experimental evaluation designs (50). This is why a significant part of the support was focused on ICPs' elicitation of their underlying theory of change, as a way for them to evaluate how and why their project work by analyzing diverse data and taking into account the context. The theory of change of each ICP should have provided them with an analytical framework, a way of making sense of diverse empirical observation. However, experimental evaluation and RCT-like design are so prevalent in the healthcare domain, that is, really difficult for some actors to get used to a different evaluation paradigm. Many involved healthcare workers wanted to do statistical comparison (before-after or with a control group) to prove the effectiveness of a specific intervention, even though their projects entail complex social processes and there are too many contextual factors at micro-, meso-, and macro-levels to allow for a robust comparison (51).

### Complex positioning of the support team

The scientific team in charge of supporting ICPs was also mandated by the government to do an external evaluation of the pilot projects assessing whether they had reached their goals and achieved integrated care and better results for quadruple aim. This double mission raised worries among ICPs' partners, and it took time to build trust, an essential precondition for the support mission, between FAITH.be team and the ICPs (52). To build this trust relation, we explained thoroughly and repeatedly our stance on evaluation to ICPs members: As the development of integrated care in Belgium was still at an early stage, our work was grounded in a developmental evaluation paradigm rather than summative evaluation (51, 53). Developmental evaluation “is used to inform adaptive development of change initiatives in complex environments” (54). The goal has been to learn from ICPs' implementation experiences, to understand why and things work in a certain context, and to build this knowledge directly with the stakeholders. Pilot projects should then be considered as partners of the evaluation process, rather than the objects of an external evaluation focused on compared effectiveness. Support and evaluation were designed to generate a virtuous circle of learning among stakeholders. In this sense, pilot projects are sites of knowledge production and we, as evaluators, sometimes defended the ICPs in front of the authorities when they exerted pressure to go faster in the implementation. Yet, adhering to developmental evaluation requires that every involved actor adopts a learning posture. This proved difficult for some policymakers who expected the evaluation to be summative, that is, to identify which ICPs work best, and which ICPs run poorly, as a mean to make decision about funding and expansion. However, as we made it clear that the support mission was not subordinate to evaluation but two faces of a same coin, ICPs understood they had more to gain than to lose. Consequently, we were able to build a fruitful collaboration with most ICPs.

### Fitting the support to a variety of needs and initial capacities

If all ICPs eventually developed self-evaluation practices and understand its potential added value, the disparities between the 12 ICPs have been a challenge all along for the support mission. When we met with each ICP individually, we had to adapt our interventions ICPs' specificities, initial capacities, and expertise within the team of coordinators and decision makers. In relation with the teams' composition and its members' education, there was a strong variance in vision and capacity regarding evaluation. The main adaptation was the intensity and frequency of the delivered support, as ICPs with a lower initial evaluation capacity were in need of more accompaniment and explanations, while those with a stronger initial capacity did not interact as much with the scientific team. Some ICPs were more prone to quantitative methods, while other were open to qualitative or mixed designs; some teams included professionals with a social science background, while others gathered only healthcare providers. The diversity of backgrounds within a team, and especially the presence of social science graduates along healthcare workers and healthcare organization managers, was a driver for a stronger evaluation capacity and for facilitated contacts with FAITH.be, as many of FAITH.be researchers came from social sciences and public health themselves: Involved social scientists facilitated dialog and the translation of concepts to the realm of the projects. The involvement of hospital data managers was also a strength, as they understood the relation between data, indicators, and measurement of a result. Yet, some ICPs' teams with a stronger background in summative, quantitative evaluation were less open to FAITH.be evaluation vision and methods, as they already adhered to other evaluation paradigms. Conversely, some teams with a very low initial evaluation capacity were very eager to learn and responded well to our support work.

### Experience-based learning as a driver

Furthermore, some evaluation concepts and methods are easier to share than other. For example, the idea of PDSA cycle is easy to understand, but the notion of theory of change and its practical implication for evaluation (choice of indicators, interpretation) is not. We quickly adapted our work, not to simply explain theoretical aspects, but to actually experiment what it means in practice, hence the use of exercises and serious games with actual data, with the ICPs' members. Practical experience, that is, learning by doing, is highlighted in the literature as a key driver to learn and to develop evaluation capacity at individual and organizational level (13, 55, 56). Experience-based learning about evaluation practice and challenges also occurred in the frame of the learning community or spontaneously: Repeated discussions between coordinators of all Belgian ICPs have allowed them to learn from each other's experiences. Sharing experience among stakeholders should be encouraged, especially as these exchanges can continue once the support mission has ended. A vivid learning community can only be achieved if there are numerous occasions for stakeholders to exchange, to get to know one another a little, and to understand they face the same challenges in many ways. It is also key for the participants to trust one another and the process, to be able to discuss more sensitive topics like difficulties and failures that may happen in ICPs. Another precondition for experience-based learning and knowledge sharing among practitioners is it to have enough time: Developing and maintaining a learning community needs time. This has been one of the main challenge for our support mission.

### New projects need time to experiment and learn

Developing integrated care through ambitious pilot projects in a context characterized by systemic fragmentation requires a lot of time (57). Yet, Belgian ICPs were funded for 4 years and the scientific team had to stop its evaluation and capacity building work after only 2.5 years of implementation. Moreover, the work of FAITH.be was riddled with administrative hurdles to build the dashboard, which entailed a heavy workload for the support team. For the ICPs, the first year of the projects has mostly been dedicated to network and governance building, as various care providers had to learn to work together and to understand what was expected from their engagement. However, ICPs' teams felt overburdened, due to, among other things, permanent demands for accountability by the federal authorities. As a result, they had only a very limited time to invest in self-evaluation practices. Moreover, evaluation is only relevant if there is something to evaluate. The provided time frame has been too short to conceptualize, implement, and evaluate a set of innovative actions and go through repeated PDSA cycles; ICPs need enough time to experiment with and learn from care integration and its evaluation. A long-term, secure perspective should be provided to achieve this—but it does not mean there should not be any evaluation early on: Early evaluation can focus on the relevance of the action, the coherence of the action plan regarding population needs, and the first year of such projects is also the right moment to plan evaluation questions, methods, and indicators for the future (e.g., which outcomes should be measured and how). Even though the scientific support funding was stopped early in the development of IC, some members of FAITH.be have kept working with the ICPs and are developing online teaching modules that ICPs' teams can use indefinitely to train themselves and understand some key concepts. This is especially relevant because there is some turnover within ICPs.

### Co-construction of accountability mechanisms

ICPs' teams complained about the permanent, time-consuming demands for accountability emanating from the authorities, without clear added value for them. However, accountability process can either hinder or facilitate evaluation practices within a project (58). Accountability mechanisms should be framed collectively by funding authorities and projects as a shared way to learn about implementation processes and results, and not simply as a mean of controlling or ranking projects in terms of “performance.” Moreover, the aim of accountability should be rooted in a common vision of what constitutes care integration and what are the building blocks to achieve care integration, based on evidence and not only on political agendas (18).

Finally, we promoted a pragmatic approach regarding data collection, as this can represent a heavy workload: Evaluation practices should not take too much time within projects, as their primary goal is to develop and implement actions to integrate care. The absence of an operational dashboard has been a huge obstacle for the support mission, as one of the main evaluation tool gathering several indicators was not available for ICPs. It is essential, in future scientific support mission, to ensure that the systems linking different databases and providing indicators for projects' self-evaluation are operational, as the availability of such indicators is one cornerstone of evaluation practices and of quality culture.

## Conclusion

### Synthesis of lessons learned and recommendations

This article provides a reflexive return on the development of scientific support for ICPs and its implementation during 2.5 years. We developed a diversified set of tools and methods to enhance evaluation capacity within interdisciplinary teams in charge of managing and implementing vast action plans. Our key messages are the following:

- The design and preparation of the support is an essential phase during which the ability to provide the various methods and tools should be assessed. In our case, the absence of a functional dashboard has been a major setback that could have been prevented if there had been a clear discussion of the availability of human and technical resources, and shared goals between the different involved parties.
- The support works best when it is co-constructed by the scientific team and the beneficiaries, as a mean to adapt the interventions to the needs and to the local context of each project. Yet, support to pilot projects is also vastly influenced by the public administrations and authorities that define the projects' funding, objectives, and accountability mechanisms: The politico-administrative context can deter or encourage experimentation and learning.
- To develop organizational evaluation capacity, a long-term, secure perspective should be provided for all stakeholders. This is a necessary condition for everyone to adopt a learning posture.

#### Strengths and limitations

This article presents practices and reflections that are based on our experience with pilot projects and policymakers during 2.5 years. The issues raised are not frequently discussed in the healthcare systems and integrated care literatures. It is limited to a description of our work and a reflexive return on it, as we could not perform a dedicated research systematically measuring the impact of our support intervention over time, with a specific methodology and clear goals from the start. Our work rather pertains to action research and developmental evaluation. It contributes to strengthening the links between evaluation capacity building literature and health system transformation involving care providers and services. We did not use an established, overarching theoretical framework to compare our intervention to a series of predefined dimensions. Indeed, to develop our support mission and to draft this article, we drew inspiration from a variety of sources originating from the evaluation literature, the integrated care literature, the quality management literature, and the gray literature. This absence of a dedicated theoretical framework can be considered as a limitation, but it is also a strength, as the work we performed actually rests on the previous experiences and expertise of the interdisciplinary scientific team.

#### Perspectives opened by this research

This article opens new perspectives; as further research is needed to assess whether the 12 ICPs still benefited from the support work a few years after the end of the mission. Specifically, it would be interesting to appraise each ICPs' evaluation capacity and to further analyze their practices and vision. More specifically, one could analyze how the four different tools and methods are being used now, and how these tools and methods help ICPs to develop further their quality culture. This analysis could interestingly check whether the main lessons and outcomes align with one or multiple existing theoretical framework within evaluation literature, capacity building literature, or implementation science literature, as a mean to confirm, or further refine these theoretical developments. Overall, and in connection with broader evaluation literature, it would be interesting to try to develop some systematic guidelines and recommendations about how to support pilot projects aiming a whole system change and adopting a population health management approach regarding their evaluation capacity.

## Author contributions

NC and BP were the co-supervisor of the support mission delivered to the integrated care pilot projects and took the initiative to draft the present article. JM contributed as the director and general supervisor of the FAITH.be consortium. All authors made substantial contributions to the design of the research project and took an active role in the support mission and in the evaluation and participated in the editing and critical revision of the manuscript and approved the final version of the manuscript for publication.

## Funding

This research has been ordered by the Federal Government of Belgium and funded by the National Institute of Health and Disability (NIHDI).

## Conflict of interest

The authors declare that the research was conducted in the absence of any commercial or financial relationships that could be construed as a potential conflict of interest.

## Publisher's note

All claims expressed in this article are solely those of the authors and do not necessarily represent those of their affiliated organizations, or those of the publisher, the editors and the reviewers. Any product that may be evaluated in this article, or claim that may be made by its manufacturer, is not guaranteed or endorsed by the publisher.
